# Potential for Nitrogen Fixation in the Fungus-Growing Termite Symbiosis

**DOI:** 10.3389/fmicb.2016.01993

**Published:** 2016-12-15

**Authors:** Panagiotis Sapountzis, Jane de Verges, Kathrin Rousk, Magdeleen Cilliers, Barend J. Vorster, Michael Poulsen

**Affiliations:** ^1^Centre for Social Evolution, Section for Ecology and Evolution, Department of Biology, University of CopenhagenCopenhagen, Denmark; ^2^Section for Terrestrial Ecology, Department of Biology, University of CopenhagenCopenhagen, Denmark; ^3^Department of Plant Production and Soil Science, Forestry and Agricultural Biotechnology Institute, University of PretoriaPretoria, South Africa

**Keywords:** macrotermitinae, *Macrotermes*, nitrogenase, *nifH*, *Odontotermes*, symbiosis

## Abstract

Termites host a gut microbiota of diverse and essential symbionts that enable specialization on dead plant material; an abundant, but nutritionally imbalanced food source. To supplement the severe shortage of dietary nitrogen (N), some termite species make use of diazotrophic bacteria to fix atmospheric nitrogen (N_2_). Fungus-growing termites (subfamily Macrotermitinae) host a fungal exosymbiont (genus *Termitomyces*) that provides digestive services and the main food source for the termites. This has been thought to obviate the need for N_2_-fixation by bacterial symbionts. Here, we challenge this notion by performing acetylene reduction assays of live colony material to show that N_2_ fixation is present in two major genera (*Macrotermes* and *Odontotermes*) of fungus-growing termites. We compare and discuss fixation rates in relation to those obtained from other termites, and suggest avenues of research that may lead to a better understanding of N_2_ fixation in fungus-growing and other termites.

## Introduction

The success of termites has been attributed to their ability to consume nutritionally imbalanced food sources, allowing them to exploit otherwise inaccessible niches (Brune, 2014). Since the first observations of termites surviving on pure cellulose by Cleveland (1925), biologists have explored the mechanisms through which termites overcome the two inherent problems of this diet: the decomposition of plant-cell walls and the acquisition of sufficient nitrogen (N) (Breznak, 1982; Higashi et al., 1992). Through decades of study, and with the advent of modern molecular methods, we now know that termites are obligately associated with symbiotic microorganisms – mainly gut bacteria – that make this possible (Bignell, 2000; Brune and Ohkuma, 2010). Termite gut microbes provide the enzymes needed to degrade plant polymers, synthesize amino acids, recycle nitrogenous waste, and fix atmospheric nitrogen (N_2_) (Benemann, 1973; Breznak et al., 1973; Potrikus and Breznak, 1981; Bentley, 1984; Bignell, 2000; Brune and Ohkuma, 2010).

**FIGURE 1:**
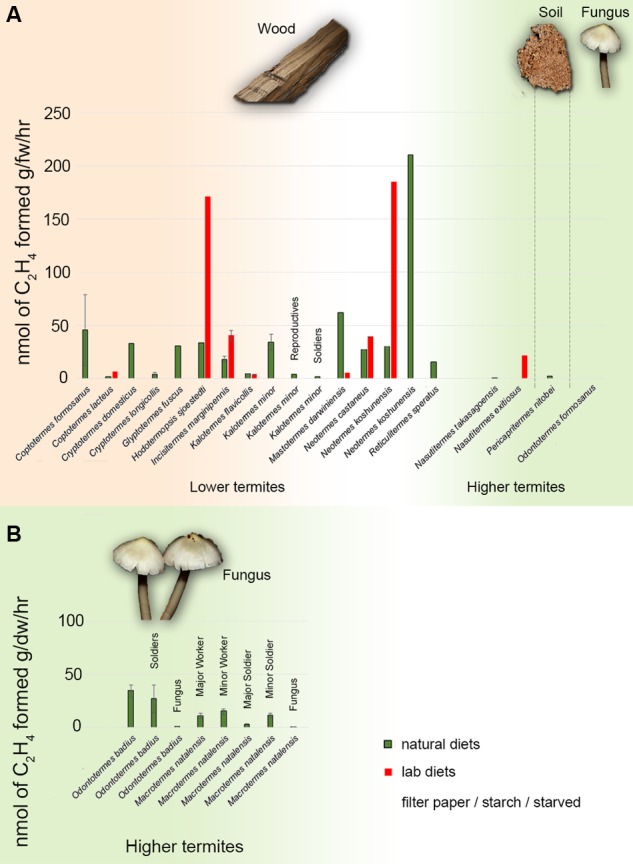
**ARA fixation rates in termites. (A)** Summary of diazotrophic fixation rates in investigated termite species from the literature. The fixation rates are expressed as nmol C_2_H_4_ per gram fresh weight per hour determined through ARA. The data presented are from Benemann (1973), French et al. (1976), Noda et al. (1999), Ohkuma et al. (1999, 2015) and Desai and Brune (2012). **(B)** ARA fixation rates measurements of fungus-growing termites from the present study; bars represent the mean of six replicates (three per colony for two colonies). Fixation rates are expressed as nmol C_2_H_4_ per gram dry weight per hour. Bar colors indicate whether the termite were fed on natural (green) or artificial (red) diets during ARA measurements. Each termite species’ natural diet is presented on top of the histograms. All measurements were performed on termite workers except where indicated above the bars. Error bars showing SE are presented, where available.

Assistance from termite gut microbes in the degradation of plant polymers has received substantial attention (Brune, 2014). In contrast, the role symbionts play in balancing the N economy – called ‘the second major symbiosis in termites’ by Higashi et al. (1992) – has not been approached in a consistent manner, and the relative importance of symbiotic diazotrophs (N_2_ fixing bacteria) in termite feeding on different substrates remains unclear (Higashi et al., 1992; Eggleton and Tayasu, 2001; Brune and Ohkuma, 2010). Termites are commonly separated into two broad categories: those that nest in and feed on a single source of dead plant material (e.g., felled dead wood; one-piece nesters) for the entire lifespan of the colony, and those that forage outside the nest (two-piece nesters) (Hidaka et al., 1987; Higashi et al., 1992). The one-piece nesters represent the ancestral lifestyle of termites, and the progressive separation of food and nest (two-piece) is evolutionarily derived (Yamada et al., 2007; Nalepa, 2015). The severe shortage of N in the diet of one-piece nesting termites (as low as 0.03% in dead wood; Breznak, 1982) necessitates supplementary N acquisition, and the association with symbiotic gut diazotrophs is thus ancestral in termites (Dietrich et al., 2014; Nalepa, 2015). Since the 1960s, evidence for such associations has accrued through testing for N_2_ fixation activity using the acetylene reduction assay (ARA), and for the incorporation of atmospheric N_2_ in termite biomass through stable isotope analyses (for a review, see Breznak, 2000; **Figure 1A**).

The fungus-growing termite sub-family Macrotermitinae, including the major genera *Macrotermes* and *Odontotermes*, are abundant in sub-Saharan Africa, and associate with basidiomycete *Termitomyces* fungi, which grow on a medium of termite feces (the fungus comb). The fungus has been suggested to provide the means to compensate for the C:N imbalance through concentration of N and selective elimination of C via respiration (Eggleton and Tayasu, 2001; Nobre et al., 2011), which could potentially explain the much higher N-content of fungus material, which has been estimated in e.g., *Macrotermes natalensis* to be approximately 7% (Rohrmann, 1978). As a result, N_2_ fixation has been thought to be absent or insignificant in fungus-growing termites, as their access to N is hypothetically nutritionally less constrained than that of other termites (Higashi et al., 1992; Eggleton and Tayasu, 2001). Considering those hypotheses but also recent work suggesting that different castes of fungus-growing termites may have different nutritional requirements as they have distinct diets and gut microbiota (Hongoh et al., 2006), we examined whether symbiotic N_2_-fixation takes place in fungus-growing termites by performing ARA on two termite species, *M. natalensis* and *Odontotermes badius.* We compare our findings with previous work on members of other termite sub-families and suggest that diazotrophic fixation should be considered present also in this sub-family.

## Materials and Methods

Termites and comb material from two colonies of *O. badius* and two colonies of *M. natalensis* were collected in January and February 2015 and maintained in the dark at room temperature at the Forestry and Agriculture Biotechnology Institute^1^, University of Pretoria. Six to ten individuals per caste were placed in triplicate 45 mL glass vials stoppered with rubber septa and containing moist filter paper for ARA. In *M. natalensis*: major workers, minor workers, major soldiers, and minor soldiers were assayed separately; in *O. badius*: major and minor workers were assayed together, and the single soldier caste was assayed separately. A further three vials contained fungus comb pieces (∼0.5 g) without termites. Four milliliter headspace atmosphere was removed using a gas-tight syringe and replaced with an equal volume of acetylene, resulting in an atmosphere containing approximately 10% acetylene. Vials were subsequently incubated at room temperature for 2 h, after which a 6 mL gas sample was removed and stored in evacuated vials (Labco, Ceredigion, UK) including three vials for negative controls (without addition of acetylene). Gas samples were analyzed by gas chromatography (GC) at the Plant Production and Soil Science Department, University of Pretoria as follows: ethylene production was measured by extracting 1 mL of gas from the headspace of each flask in a gas chromatograph (GC 2025; Shimadzu, Japan) according to the method described by Turner and Gibson (1980). A flame ionization detector was used with an oven temperature of 200°C, column temperature of 130°C, a gas flow of: air (400), H_2_ (40), He carrier gas (40) and running time was 3 min. Ethylene peak areas were calibrated against a standard curve calculated from pure (99.9%) standards of known concentration. Following the experiment, samples were dried at 60°C for 48 h and weighed. Ethylene areas were converted to nmol C_2_H_4_/g dw/hr (gram dry weight per hour). Since gas samples were taken at a single time point, C_2_H_4_ values from ARAs represent an estimate within a given fixation range; this, and possible differences between our methods and those in previous works, precluded statistical analyses.

## Results

Positive AR activity was detected in all colonies (**Figure 1B**). Ethylene was not detected in the negative controls of live termites in the absence of acetylene. In *M. natalensis*, the highest AR activity was in major workers, minor workers and minor soldiers, while major soldiers showed lower activity and the fungus comb showed the lowest activity (**Figure 1B**). In *O. badius*, the highest activity was found in workers and then in soldiers, while the fungus comb showed very low AR activity (**Figure 1B**).

## Discussion

For almost a century it has been hypothesized that termites may acquire their necessary N through microbial N_2_ fixation (Cleveland, 1925; Peklo, 1946). Early work using the ARA demonstrated that N_2_ fixation is present (Benemann, 1973; Breznak et al., 1973), that rates are highest in workers, and that fixation is performed by gut bacteria (French et al., 1976). Several termite species are able to acquire atmospheric N_2_ with the help of N_2_-fixing bacteria (Täyasu et al., 1994; Desai and Brune, 2012), including both lower and higher termites (**Figure 1A**). Since the necessity for N_2_ fixation arises from the N-poor diet, N_2_ fixation can be suppressed when termites are fed on N-rich diets (Breznak et al., 1973; Meuti et al., 2010; Desai and Brune, 2012), a pattern also observed in a comparative study (He et al., 2013) between the dung-feeding (N-rich) *Amitermes wheeleri* and the wood-feeding (N-poor) *Nasutitermes*: the latter with an overrepresentation of nitrogenase genes and a higher abundance of one of the major N_2_ fixing taxa in termites, the Spirochaetes.

We found positive, caste-specific AR activity in live *M. natalensis* and *O. badius* termites, but almost no activity in the fungus comb (**Figure 1B**). This supports that fixation takes place within the termite gut and not in the external fungus comb. The higher fixation rates in workers than soldiers is consistent with the fact that workers consume the foraged plant material and fungus comb, while soldiers and larvae are trophically dependent, relying on nutrition via proctodeal trophallaxis from workers (Eggleton, 2010). These differences in feeding strategy and consequently symbiotic roles of the gut bacteria has been shown in *Macrotermes gilvus* to be reflected in the composition of the gut microbiota, which cluster more by termite caste and age than by colony (Hongoh et al., 2006).

Even if diazotrophic AR rates in fungus-growing and wood-eating termites are not quantitatively comparable, mainly because previous studies quantified rates per gram of fresh while we used dry material, AR rates in fungus-growing termites are likely to be lower than what has been reported in wood-eating termites (**Figure 1**). Nevertheless, although fungus-growing termites may be less nutritionally constrained by their lifestyle than other termites (Brune and Ohkuma, 2010), this does not rule out that N_2_ fixation may be functionally important, although the contribution of N_2_ fixation to the N budget remains to be quantified. This challenges the notion that N_2_ fixation should not be important in fungus-growing termites that has arisen, because the fungal diet obviates the need for costly fixation (Breznak, 1982). Fungus-growing termites have been proposed to rely on methanogenesis by gut Archaea and respiration by the fungus comb to eliminate carbon (C); contributing to balance the high C:N-ratio of their forage (Higashi et al., 1992; Eggleton and Tayasu, 2001). In a recent metagenomic analysis in *Odontotermes yunnanensis* the authors did not manage to amplify *nifH* genes (Liu et al., 2013). Similarly, a previous study in *Odontotermes formosanus* found only few functional *nifH* genes compared to those found in wood-eating termites (Yamada et al., 2007), and these were believed to belong to a “pseudo” *nifH* clade (Ohkuma et al., 1999). The latter finding has, however, been challenged by recent work (Zheng et al., 2016). Thus, targeted work to elucidate the presence and expression of *nifH* and the responsible producers is needed.

It has been suggested that the bacteria responsible for N_2_ fixation in termites are often abundant gut bacterial taxa in the Bacteroidetes, Spirochaetes, and Clostridia (Ohkuma et al., 1996, 1999; Lilburn et al., 2001; Warnecke et al., 2007; Yamada et al., 2007; Burnum et al., 2011; Du et al., 2012) (reviewed in Brune, 2014), which indeed are abundant in fungus-growing termite guts (Otani et al., 2014; Poulsen et al., 2014; reviewed in Brune, 2014). The high diversity of *nifH* genes or transcripts discovered in some of the above studies (Ohkuma et al., 1999; Yamada et al., 2007), and previous work, indicates that other gut bacterial taxa may also contribute (Potrikus and Breznak, 1977; Doolittle et al., 2008; Isanapong et al., 2012; Wertz et al., 2012; Zheng et al., 2016). In addition, the lower termites harbor unique flagellated protists in their guts that play a key role in host nutrition; these protists harbor bacterial endosymbionts (Hongoh et al., 2008; Ohkuma et al., 2015) or ectosymbionts (Desai and Brune, 2012) that may also fix N_2_.

To obtain a more fundamental understanding of N_2_ fixation in fungus-growing and other termites, as **Figure 1** documents the generally fragmented efforts, we propose that future work should seek to: (i) thoroughly characterize the responsible diazotrophs across termites, including fungus-growing and soil feeding termites, where fixation has also been proposed to be less important (Breznak, 1982; Ohkuma et al., 1999); (ii) couple N_2_ fixation rates with detailed trophic habits of different termite castes, as fixation rates are likely to depend on differences in diets due to differences in food sources and nutritional requirements, as indicated by our current findings (**Figure 1B**); (iii) investigate differences in fixation rates using N-isotopes rather than AR, which would be more suitable for functional comparisons and quantification of the importance of incorporation of N; (iv) cultivate, when possible, N_2_-fixing bacteria from different termite species, castes, and ages to evaluate their activity *in vitro*, and (v) better incorporate culture-independent methods such as metagenomics to allow for characterisation of nitrogenase genes in gut bacteria and advanced microscopy approaches to pinpoint gut compartment where N_2_ fixation takes place (cf. Warnecke et al., 2007; Sapountzis et al., 2015).

## Author Contributions

PS and MP designed the project and supervised JdV, who took part in field collections and performed the experiments with help from MC (supervised by BV) and KR. All authors contributed to writing the manuscript.

## Conflict of Interest Statement

The authors declare that the research was conducted in the absence of any commercial or financial relationships that could be construed as a potential conflict of interest.

## Footnotes

1 http://www.fabinet.up.ac.za/
